# Lead Monoxide Nanostructures for Nanophotonics: A Review

**DOI:** 10.3390/nano13121842

**Published:** 2023-06-12

**Authors:** Hongyan Chen, Mengke Wang, Weichun Huang

**Affiliations:** 1 Engineering Training Center, Nantong University, Nantong 226019, China; chenhy@ntu.edu.cn; 2 School of Chemistry and Chemical Engineering, Nantong University, Nantong 226019, China; huangweichun@ntu.edu.cn

**Keywords:** lead monoxide, black phosphorus analogs, composites, ultrafast fiber laser, nanophotonics

## Abstract

Black-phosphorus-analog lead monoxide (PbO), as a new emerging 2D material, has rapidly gained popularity in recent years due to its unique optical and electronic properties. Recently, both theoretical prediction and experimental confirmation have revealed that PbO exhibits excellent semiconductor properties, including a tunable bandgap, high carrier mobility, and excellent photoresponse performance, which is undoubtedly of great interest to explore its practical application in a variety of fields, especially in nanophotonics. In this minireview, we firstly summarize the synthesis of PbO nanostructures with different dimensionalities, then highlight the recent progress in the optoelectronics/photonics applications based on PbO nanostructures, and present some personal insights on the current challenges and future opportunities in this research area. It is anticipated that this minireview can pave the way to fundamental research on functional black-phosphorus-analog PbO-nanostructure-based devices to meet the growing demands for next-generation systems.

## 1. Introduction

In recent years, a new family of materials which have similar folded structures to black phosphorus, termed black phosphorus analogs, not only show a tunable bandgap, superior on/off ratio, and carrier mobility, but also exhibit impressive environmental stability [1,2,3,4,5,6,7,8,9] and hold great promise in practical applications. Until now, a number of reviews on black phosphorus analogs, such as bismuthene [4,10,11], antimonene [12], tellurene [13,14], etc., have been systematically summarized due to their fascinating properties. For example, our group recently summarized the controlled synthesis of monoelemental bismuth (Bi) nanomaterials with different shapes and sizes, and highlighted the recent advances of their intriguing applications [4]. In 2018, Ares et al. [12] reviewed the different approaches and characterization techniques for antimonene and summarized the electronic band structure of antimonene for optoelectronics and thermoelectric applications in both monolayer and few-layer forms. In 2017, Yu et al. [14] reviewed the controlled synthesis of tellurium (Te) nanostructures and summarized the latest progress in many applications, such as batteries, photodetectors, sensors, etc.

Apart from that, black-phosphorus-analog lead monoxide (PbO) is also of research interest in recent years owing to its excellent photodetection and nonlinear optical properties [15,16,17,18,19,20]. PbO is a layer compound which exists in two crystal forms: red tetragonal (α) and yellow orthorhombic (β) forms [21], both of which have been experimentally synthesized [20,22,23,24]. Due to the same folded structure as that of black phosphorus for the yellow orthorhombic crystal form, more attention was paid to β-PbO to pursue high-performance black-phosphorus-analog-based devices. The unique properties of PbO nanostructures for practical applications are predominantly dependent on their structural stability, as well as their electronic and spintronic characteristics. Moreover, the low cost, easy fabrication, and high environmental stability of PbO nanostructures are also of significance to provide them with a great potential in a variety of applications, such as CO_2_ photoreduction [25], gas detection [26], batteries [27], supercapacitors [28], and photovoltaics [29].

**Figure 1:**
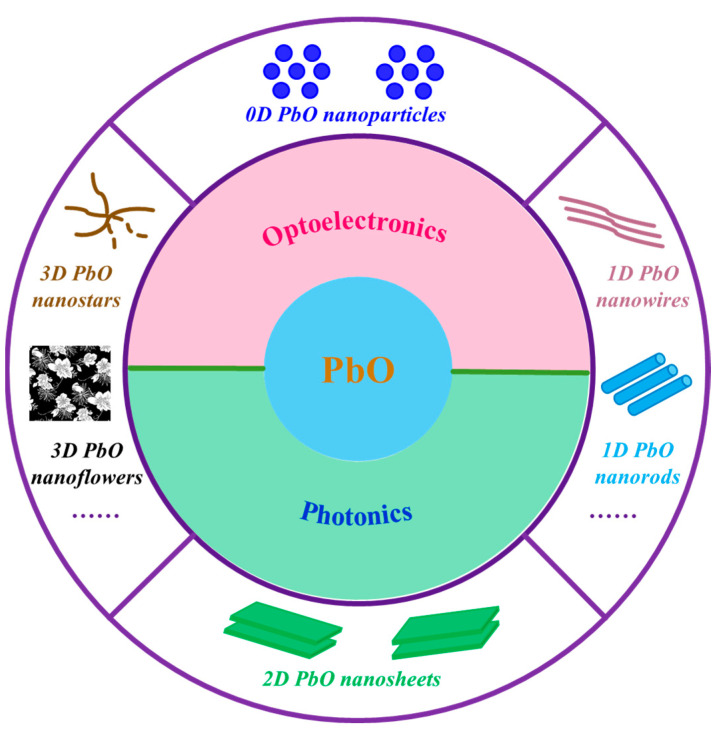
Schematic illustration of the morphology control of PbO nanostructures and their optoelectronics/photonics applications.

Inspired by the recently published reviews on black phosphorus analogs, such as bismuthene [4,11], antimonene [12], and tellurene [13,14], we summarize and highlight the recent progress in the elaborate design and controlled synthesis of PbO nanostructures, their fascinating properties, and emerging applications, as shown in Figure 1. We firstly summarize PbO nanostructures with different dimensionalities, including zero-dimensional (0D) nanoparticles (NPs), one-dimensional (1D) nanostructures (nanowires (NWs), nanorods (NRs), etc.), two-dimensional (2D) nanosheets (NSs), and three-dimensional (3D) Bi-based nanostructures. Afterwards, the fundamental properties and optoelectronic/photonic performance of PbO nanostructures are reviewed. Last, but not least, the challenges and opportunities in future high-performance nanophotonic devices based on PbO-related nanostructures are discussed.

## 2. Synthesis of PbO Nanostructures

In recent years, the controlled synthesis of PbO nanocrystals in a series of shapes, including 0D NPs, 1D nanostructures (e.g., NWs, NRs, etc.), 2D NSs, and even 3D hierarchical nanostructures, has achieved significant progress. The synthetic methods, such as liquid-phase exfoliation (LPE) and hydrothermal or solvothermal methods, are generally divided into two categories: the “top-down” strategy and the “bottom-up” strategy. The “top-down” strategy is usually used to exfoliate layered bulk PbO crystals into 0D NPs or 2D single- or few-layered NSs under driving forces, such as sonication and scotch tape, due to their weak van der Waals interaction between neighboring stacked layers. The typical “top-down” strategies include LPE and mechanical cleavage. Note that the “bottom-up” strategy usually depends on the rational design and controlled synthesis of PbO nanostructures from the precursors under certain conditions, while the typical “bottom-up” technique for preparing high-quality PbO nanostructures is the hydrothermal method or the solvothermal method. In this section, the PbO nanostructures with different dimensionalities are briefly summarized. It is noted that only the widely used approaches are presented in this section rather than all the synthetic approaches.

### 2.1. Zero-Dimensional PbO NPs

**Figure 2:**
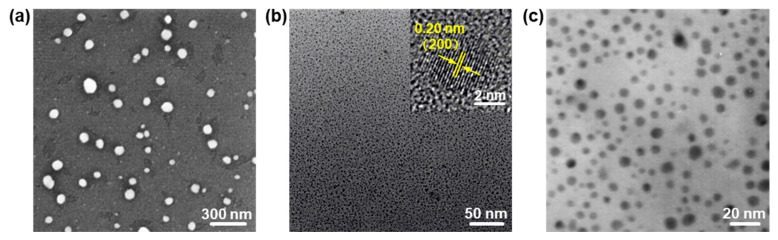
The structural characterizations of 0D PbO NPs. (**a**) SEM image of PbO NPs formed by laser ablation. Reproduced with permission from Reference [42]. Copyright 2019, Elsevier B. V. (**b**) TEM image of PbO QDs prepared by an LPE method (inset showing the HRTEM image of one single PbO QD). Reproduced with permission from Reference [20]. Copyright 2018, The Royal Society of Chemistry. (**c**) TEM image of PbO NPs formed at the water/air interface with the subphase concentration of 1 × 10^−3^ mol L^−1^ at 30 °C. Reproduced with permission from Reference [44]. Copyright 2011, Elsevier B. V.

NPs (with a size range from 1 nm to 100 nm) have attracted extensive attention because of their versatile applications in (opto)electronics, catalysts, sensors, and biomedicines [3,5,6,7,20,30,31,32,33,34,35,36,37,38,39,40,41]. In 2019, Shur et al. [42] reported the formation of PbO NPs by laser ablation in hot water using Pb as a model metal. The PbO nanoparticles in a classical spherical shape can be observed immediately after only the laser ablation treatment (Figure 2a), and the spherical NP shape rapidly changes to octahedra, rods, or plates when heated by laser ablation in water. Additionally, in 2018, our group successfully fabricated PbO quantum dots (QDs) with an LPE method [20]. The transmission electron microscopy (TEM) of the as-prepared PbO QDs showed an average lateral size of 3.2 ± 0.9 nm and an average thickness of 2.5 ± 0.5 nm, which corresponds to 4 ± 1 layers (Figure 2b). The high-resolution TEM (HRTEM) image of the PbO QDs displays a clear lattice fringe of 0.20 nm (Figure 2b inset), which is well assigned to the (200) plane of the β-PbO crystal [43]. Moreover, Chen et al. [44] demonstrated that PbO NPs were successfully synthesized at the water/air interface in the condition of the Langmuir films of poly(N-vinylcarbazole). A large quantity of round PbO NPs with a diameter of several nanometers can be observed (Figure 2c). It should be pointed out that, if the temperature rises up to 40–50 °C, the size of the as-prepared NPs sharply increases and the crystallinity also improves [44].

### 2.2. One-Dimensional PbO Nanostructures

**Figure 3:**
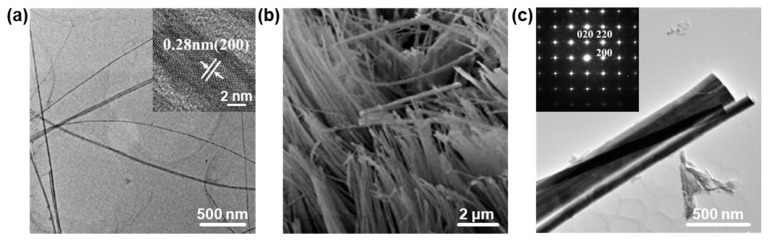
The structural characterizations of 1D PbO nanostructures. (**a**) TEM image of ultrathin PbO NWs prepared by an alkylamine-mediated thermolysis route (inset showing the HRTEM image). Reproduced with permission from Reference [49]. Copyright 2020, Elsevier B. V. (**b**) SEM image of PbO NWs prepared by an improved sol–gel method combined with porous AAO templates. Reproduced with permission from Reference [50]. Copyright 2007, American Chemical Society. (**c**) TEM image of PbO NRs prepared by a hydrothermal method in the presence of citrate (inset showing the corresponding SAED pattern). Reproduced with permission from Reference [51]. Copyright 2006, Elsevier B. V.

The 1D PbO nanostructures are fascinating systems, similar to other 1D nanostructure systems (e.g., 1D Te NWs [14], 1D Se nanotubes (NTs) [6], 1D Bi nanobelts [45], 1D ZnO NRs [46,47], and PtCu NWs [48]), for studying physicochemical properties due to their anisotropic character. For example, a facile alkylamine-mediated thermolysis strategy was reported for the fabrication of high-quality PbO NWs through the thermal decomposition of lead carboxylate in the presence of hexadecylamine (HDA) [49]. The uniform PbO NWs, with an average length of several micrometers and an average diameter of 7.1 nm, were successfully achieved in a high yield (Figure 3a), and closer observation revealed that the as-prepared PbO NWs displayed the nature of a single crystal with a clear lattice fringe of 0.28 nm, which can be assigned to the (200) plane of the β-PbO crystal [49]. Additionally, Wang et al. [50] employed porous anodic aluminum oxide (AAO) templates to prepare a PbO NW array by a sol–gel method. The SEM image shows that the as-prepared PbO NWs were successfully grown in the nanochannels of the AAO templates (Figure 3b), and all the NWs parallelly aligned to each other, showing an excellent vertical orientation on the AAO template to form an array (Figure 3b). The average diameter of these as-prepared nanowires is ~80 nm, consistent with the channel diameter of the AAO template. Furthermore, Jia et al. [51] successfully synthesized single-crystalline PbO NRs with a hydrothermal approach. The TEM image shows that some NRs are parallel to each other, and that shorter NRs assemble beside the longer ones (Figure 3c). The SAED pattern (Figure 3c inset) shows that the rod-shaped crystal grows along the (100) direction. Because of the size-dependent effect on the performance (the absorption property and bandgap energy (*E*_g_) [17,19,20]), Bi nanostructures with different sizes can be readily synthesized by facilely tuning the reaction conditions (e.g., the reaction temperature and reaction time).

### 2.3. Two-Dimensional PbO NSs

**Figure 4:**
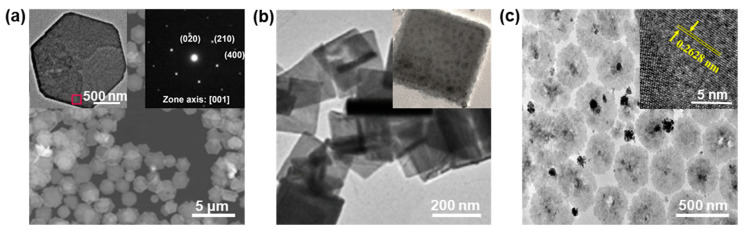
The structural characterizations of 2D PbO NSs. (**a**) Typical SEM image of PbO NSs synthesized by laser ablation in water and subsequent ambient aging (top-left inset: TEM image of an NS; top-right inset: the corresponding SAED pattern). Reproduced with permission from Reference [26]. Copyright 2020, Elsevier B. V. (**b**) Representative TEM image of PbO NSs prepared by an alkylamine-mediated thermolysis route in the presence of HDA (inset showing the corresponding magnified image). Reproduced with permission from Reference [49]. Copyright 2020, Elsevier B. V. (**c**) Typical TEM image of the PbO NSs fabricated by an LPE method showing the circle-shaped 2D NS feature (inset showing the HRTEM image). Reproduced with permission from Reference [19]. Copyright 2018, American Chemical Society.

Two-dimensional NS materials, atomically thin sheets, have attracted tremendous interest due to their fascinating properties [52,53,54,55,56,57,58]. Inspired by the huge success of graphene and black phosphorus, a series of 2D materials have been exploited and demonstrated to have great potential in many applications, such as energy [8,59,60,61], catalysis [48,62,63,64], sensors [65,66,67,68], nanophotonics [9,58,69,70,71,72], and biomedicines [73,74,75]. Although great progress in 0D and 1D PbO nanostructures has been achieved, much less is known about 2D PbO NSs, their synthetic strategies, and their fascinating performance so far. In 2020, Fu et al. [26] developed a chemically clean route for synthesizing 2D PbO NSs by laser ablation. The SEM image of the as-fabricated PbO NSs shows a mean size of 1.5 μm in the planar dimension (Figure 4a). The NS displays a hexagonal shape with a regular edge, as well as a smooth surface (left inset of Figure 4a), and the SAED pattern indicates that the NS shows a (002)-terminated surface with a single-crystal nature (right inset of Figure 4a). Moreover, in 2020, high-quality PbO NSs were also successfully synthesized by the thermal decomposition of lead carboxylate, the same as the abovementioned PbO NWs, without a higher HDA content (Figure 3c) [49]. The uniform PbO NSs, with a lateral size of ~300 nm, were successfully obtained at the initial reaction time of 30 min (Figure 4b), and typical square-like NSs are clearly observed (Figure 4b inset), indicating the successful synthesis of the 2D PbO nanostructures. In addition, in 2018, our group employed an LPE method to successfully fabricate circular PbO NSs in an isopropanol (IPA) solvent [19]. The TEM image of the circular 2D PbO NSs shows that the diameter of these as-prepared PbO NSs range from ~260 nm to ~400 nm (Figure 4c). The HRTEM image exhibits that a clear fringe lattice of ~0.263 nm can be indexed to the (200) plane of the β-PbO crystal [19]. It should be pointed out that the preparation conditions in the LPE, such as the sonication power and time, as well as the solvent and sonication temperature, have a crucial effect on the size and morphology (for NSs or NPs) of the final PbO product.

### 2.4. Three-Dimensional PbO Hierarchical Heterostructures

**Figure 5:**
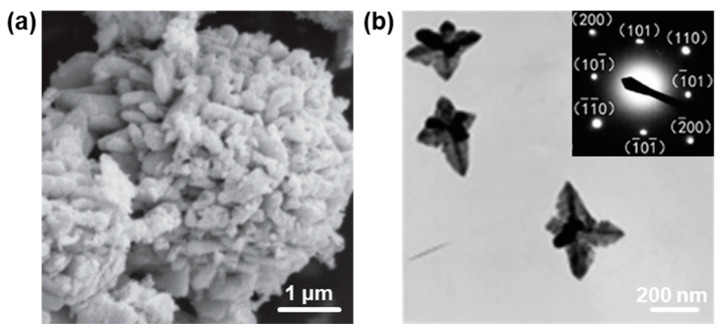
The structural characterizations of 3D PbO nanostructures. (**a**) Typical SEM image of 3D PbO nanostructures prepared by thermal deposition at 420 °C for 30 min. Reproduced with permission from Reference [83]. Copyright 2012, Elsevier B. V. (**b**) TEM image of 3D PbO nanostars formed at the air/water interface with the subphase concentration of 1 × 10^−4^ mol L^−1^ at 30 °C (inset showing the corresponding SAED pattern). Reproduced with permission from Reference [44]. Copyright 2011, Elsevier B. V.

Because of relatively easier manipulation and large-scale preparation compared to 0D, 1D, and 2D nanostructures, 3D hierarchical nanostructures have become more appealing in many fields, such as catalysis [76,77,78], sensors [79,80], optoelectronics [81,82], etc. Although low-dimensional (0D, 1D, and 2D) PbO nanostructures have achieved great progress in recent years, 3D hierarchical PbO nanostructures are also significant due to their unique properties [44,83,84]. For example, Behnoudnia and Dehghani [83] successfully synthesized 3D PbO nanostructures by the thermal decomposition of PbC_2_O_4_ nanostructures at 420 °C for 30 min, as shown in Figure 5a. Additionally, Chen et al. [44] reported that 3D PbO nanostars and nanodendrites with several hundreds of nanometers were successfully synthesized when the subphase concentration of round or irregular PbO NPs was 1 × 10^−4^ mol L^−1^. It can be seen that the as-synthesized 3D PbO nanostars contain a couple of nanodendrites, and each nanodendrite is composed of a trunk and several branches that are nearly vertical to the predominant trunk (Figure 5b). The SAED pattern shows that the as-synthesized 3D PbO nanostars display regularly arranged spots, which can be assigned to those of the β-PbO, suggesting that the as-obtained nanostars are single-crystalline.

## 3. Properties and Applications in Nanophotonics

In comparison to the bulk PbO, PbO nanostructures display intriguing properties due to the quantum confinement effect, which makes them hold great promise in the community of optoelectronics/photonics [17,18,19,20,85,86,87]. As we know, the structure–property relationship of nanostructures has a great effect on the practical application. Therefore, in the following section, the properties and the optoelectronic/photonic applications of PbO nanostructures are discussed.

### 3.1. Optical Properties

**Figure 6:**
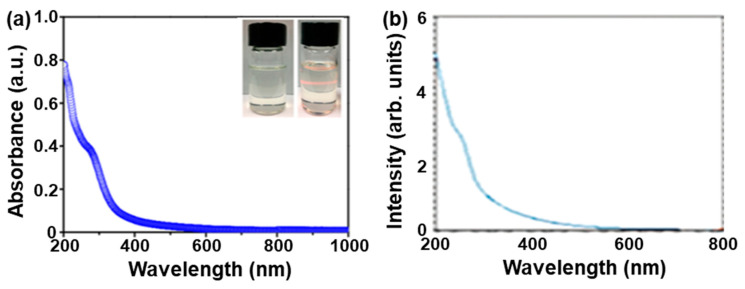
Optical properties of PbO nanostructures. (**a**) UV–Vis–NIR absorption spectrum of the 2D PbO nanosheets fabricated by an LPE method in an IPA solvent. The inset shows photos of the 2D PbO/IPA dispersion (left) and the Tyndall effect (right). Reproduced with permission from Reference [19]. Copyright 2018, American Chemical Society. (**b**) Optical absorption of PbO nanocrystals prepared by a one-pot colloidal synthesis method. Reproduced with permission from Reference [88]. Copyright 2010, The Royal Society of Chemistry.

Due to a relatively lower *E*_g_, PbO nanostructures usually show a strong photoresponse absorption in the UV–Vis spectrum [19,88]. For example, our group recently reported that the 2D PbO NSs fabricated by an LPE method exhibited a typical optical absorption (200–500 nm) (Figure 6a), indicating that they have great potential for a UV–Vis photoresponse ability [19]. Note that the obtained 2D PbO NSs can be easily redispersed in IPA and stabilized for even a half-month (left inset of Figure 6a), confirmed by the Tyndall effect (right inset of Figure 6a). In addition, Cattley and colleagues [88] reported that the PbO nanocrystals synthesized by a colloidal synthesis method displayed a typical strong absorption below 500 nm, the same as that in our previously reported PbO QDs sample fabricated by an LPE method [20].

### 3.2. Bandgap Properties

**Figure 7:**
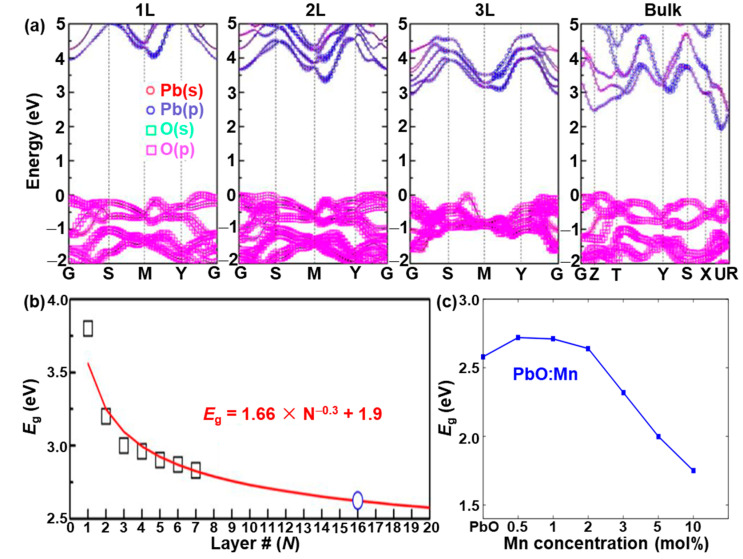
First-principles density functional theory calculations and experimental results of PbO. (**a**) Orbital-projected G0W0-corrected band structure of monolayer, double-layer, triple-layer, and bulk PbO. The VBM is dominated by the oxygen p orbitals, and the lead p orbitals take more part in the CBM region. (**b**) Relationship between the *E*_g_ and the number of layers. The estimated layer number of 16 is indicated by the blue circle. Reproduced with permission from Reference [19]. Copyright 2018, American Chemical Society. (**c**) Bandgap of Mn-doped PbO thin films with a variation in the Mn concentration, calculated based on the fundamental absorption. Reproduced with permission from Reference [89]. Copyright 2018, Elsevier B. V.

A variety of strategies, such as the density functional theory (DFT) and Tauc plots based on UV–Vis–NIR absorption spectroscopy, were performed to systematically study the electronic band structure in both monolayer and few-layer PbO. Because of the different characterization techniques, the *E*_g_s of the PbO with different thicknesses obtained from the experimental and theoretical results may have slight differences. In 2018, Xing et al. [19] used DFT calculations to demonstrate that the *E*_g_ of 2D PbO could be easily tuned by an external electric field with a huge Stark effect. The band structures of the monolayer (1L), double-layer (2L), triple-layer (3L), and bulk state of 2D PbO are shown in Figure 7a. The relationship between the *E*_g_ and layer number (*N*) of 2D PbO can be well fitted as *E*_g_ = 1.66 × *N^−^*^0.3^ + 1.9, and shows that 2D PbO has a converged *E*_g_ value as *N* reaches up to 7 (Figure 7b). It should be pointed out that the 2D PbO with an *N* ranging from 10 (2.73 eV) to 30 (2.50 eV) can absorb light with a wavelength of <500 nm, which is in good accordance with the optical result in Figure 6a. In addition, in 2018, Suryawanshi et al. [89] reported that manganese (Mn)-doped PbO films were successfully fabricated by an ultrasonic spray pyrolysis, and the *E*_g_ of Mn-doped PbO films calculated by the Tauc plot based on the absorption spectra varied with the increase in the Mn doping level. As seen in Figure 7c, there was an initial increase in the *E*_g_ for the Mn-doped PbO films with a smaller doping level of Mn, followed by a significant reduction in the *E*_g_ at a higher doping level of Mn. It can be observed that the *E*_g_ value of Mn-doped PbO films improves as the Mn doping level reaches < 2 mol% compared with that of the pure PbO film. However, with the further enhancement in the Mn doping level, the *E*_g_ value largely decreases. The *E*_g_ value has a minimum value of 1.66 eV as the Mn doping level further increases to 10 mol%, which is mainly attributed to the structural deformation in the PbO films induced by the Mn ion substituting Pb ions in the PbO lattice. This phenomenon is in good accordance with the fact that the crystallinity of PbO thin films gradually declines as the concentration of doped Mn increases [89].

### 3.3. Photodetection Performance

**Figure 8:**
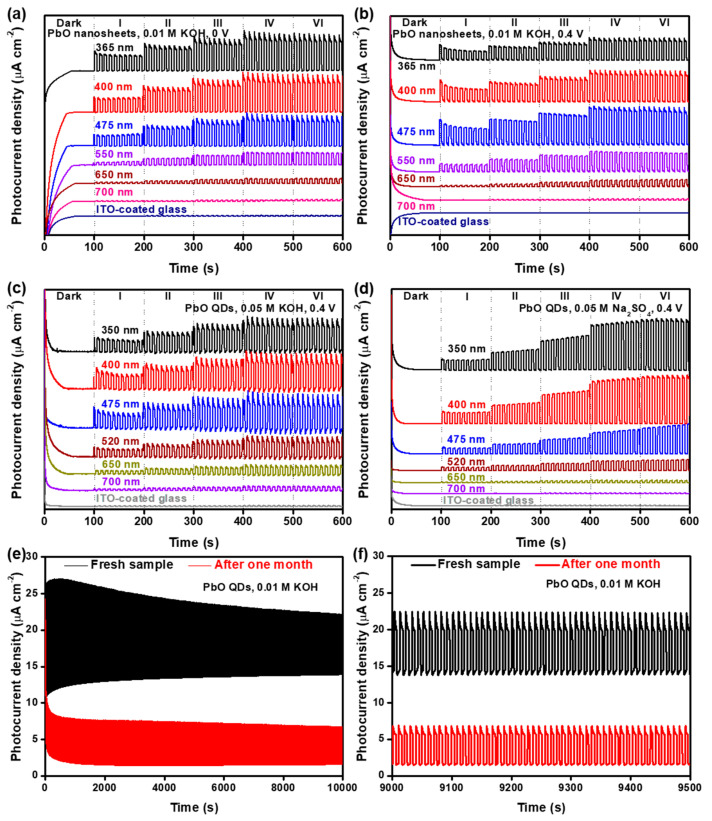
Photoresponse behavior of the as-prepared PbO nanostructures. (**a**) On/off switching behavior at voltages of (**a**) 0 V and (**b**) 0.4 V under lasers with different wavelengths (365, 400, 475, 550, 650, and 700 nm) in 0.01 M KOH. Photoresponse behavior of the PbO QD-based photodetector under lasers with various wavelengths (350, 400, 475, 520, 650, and 700 nm) at 0.4 V and various laser power densities in (**c**) 0.05 M KOH and (**d**) 0.05 M Na_2_SO_4_. (**e**) Stability of the photoresponse behavior of the PbO QD-based photodetector irradiated by simulated light in 0.01 M KOH at 0.2 V and at 118 mW cm^−2^ before and after one month, and (**f**) the selected enlarged region in (**e**). Traces are shifted vertically for clarity. Reproduced with permission from Reference [20]. Copyright 2018, The Royal Society of Chemistry.

As a narrow bandgap metal oxide, the structure of PbO is similar to that of black phosphorus, which has great potential in high-performance optoelectronic devices, such photodetectors and field-effect transistors [7,19,20]. For example, 2D PbO NSs showed excellent self-powered photoresponse behavior (Figure 8a) [19]. The 2D PbO NSs exhibited an obvious on/off switching behavior at 0 V (Figure 8a) and 0.4 V (Figure 8a), and these signals can be significantly boosted by increasing the light power, which can be attributed to the piezo-phototronic effect [90]. However, a naked indium–tin oxide (ITO) glass without 2D PbO NSs displays a negligible signal, even if the light power density has been largely augmented (Figure 8a,b), indicating that the on/off switching photoresponse behavior indeed originates from the 2D PbO NSs themselves [19]. The similar on/off switching photoresponse behavior of the as-fabricated 2D PbO-based photodetector can be easily triggered under lasers with a wavelength value of < 650 nm, which is consistent with the UV–Vis–NIR result (Figure 6a). The stronger photoresponse signal can be observed under a laser with lower-wavelength light (e.g., 365, 400, and 475 nm), which is attributed to the higher photon energy. In addition, the 0D PbO QDs similarly fabricated by an LPE method also have the same trend in the photoresponse behaviors as the 2D PbO NSs (Figure 8c,d) [20]. The photocurrent signal keeps almost stable at a wavelength of ≤400 nm and obviously declines as the wavelength rises up to >400 nm in 0.05 M Na_2_SO_4_ (Figure 8d). Such discrepancy in the KOH and Na_2_SO_4_ electrolytes can be mainly ascribed to the different functionalities between the PbO nanostructures on the substrate surface and the different types of electrolytes [20]. More importantly, the cycling stability of the PbO QD-based photodetector was traced under a simulated light in 0.01 M KOH at 118 mW cm^−2^ (Figure 8e,f). It can be observed that the PbO QD-based photodetector displays a strong on/off switching behavior, and a mere 37.2% reduction of the photocurrent density is obtained after one month of continuous measurement in 0.01 M KOH, suggesting its superior stability compared to the previously reported nanostructures, such as black phosphorus NSs [90], SnS NSs [91], and Bi QDs [34], which demonstrates that this black-phosphorus-analog PbO QD-based photodetector can shed new light on the construction of high-performance PEC-type devices.

### 3.4. Nonlinear Photonics

**Figure 9:**
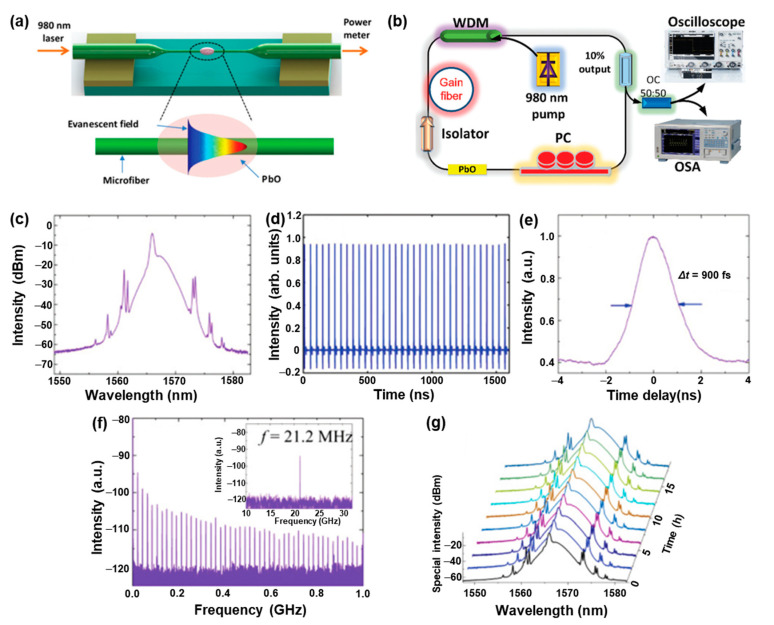
Mode-locking performance of a few-layer PbO-deposited fiber-patch-cord-based erbium-doped fiber laser. (**a**) Schematic diagram of the fabrication of a few-layer PbO-decorated microfiber. (**b**) Schematic diagram of the erbium-doped fiber ring laser mode-locked by a few-layer PbO-decorated microfiber-based saturable absorber. PC: polarization controller; EDF: erbium-doped fiber as gain medium; OC: optical coupler; BS: beam splitter. (**c**) Optical spectrum of the laser output. (**d**) Mode-locking pulse train measured by a real-time oscilloscope. (**e**) Autocorrelation trace of the pulses. (**f**) RF spectrum of the pulses. (**g**) Long-term measurement of the optical spectrum of mode-locking pulses over 16 h. Reproduced with permission from Reference [18]. Copyright 2019, The Royal Society of Chemistry.

In the past decade, nonlinear photonics based on black-phosphorus-analog nanostructures, such as Bi, Se, SnS, PbO, etc., have drawn extensive attention due to their strong spin–orbit interaction, unique electronic transport, and excellent stability [4,5,6,17,18,33,92,93,94,95,96]. For example, in 2019, Song et al. [18] reported that ultrashort pulse generation in a ring-cavity fiber laser based on a few-layer PbO could be employed as a mode-locker in erbium-doped fiber lasers. Figure 9a provides the schematic diagram of the few-layer PbO-decorated microfiber. The dispersion containing the few-layer PbO NSs as saturable absorbers (SAs) was directly deposited onto a piece of microfiber by a flame taper method [95] to form a nonlinear optical device. The few-layer PbO-based microfiber was incorporated into a fiber laser cavity (Figure 9b), and the fiber laser was reversely pumped by a 980 nm laser diode with a maximum pump power of 1.2 W to overcome the propagation of the pump light. The mode-locking activity of the as-fabricated erbium-doped fiber laser displayed a mode-locked pulse of ~1557.68 nm with a 3-dB spectral bandwidth of ~2.9 nm (Figure 9c). The Kelly sidebands in the optical spectrum suggest that the mode-locked pulses are shaped in the manner of an optical soliton. The mode-locked pulse train obtained by a real-time oscilloscope (Figure 9d) demonstrates that the repetition rate is consistent with the cavity length, verifying that the pulses indeed originate from the mode-locking function. Additionally, a commercial autocorrelator was employed to investigate the pulse duration of the mode-locked pulse, and a pulse width of ~900 fs was achieved (Figure 9e). Moreover, the stability of the pulse train evaluated by the radio frequency (RF) spectrum presents a signal-to-noise ratio (SNR) of ~50 dB, indicating that the fiber laser operation is quite stable in this system (Figure 9f). The optical spectrum of the mode-locking pulses lasting over 16 h confirms that the employed few-layer PbO NSs are capable of supporting stable mode-locking in ultrafast lasers (Figure 9e), which provide fundamental guidance on the functional ultrafast photonics based on the few-layer PbO for practical applications.

**Figure 10:**
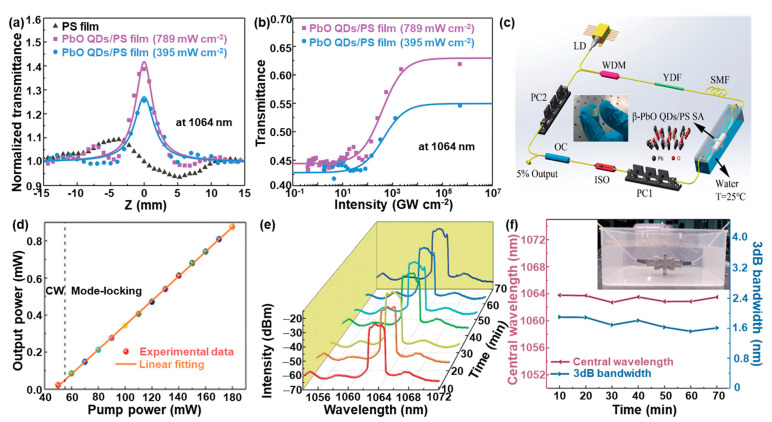
Nonlinear photonics of the PbO QD/PS-composite-based SA. (**a**) Saturable absorption experimental data obtained from the open aperture *Z*-scan technique at 1064 nm. (**b**) The nonlinear transmission curve of the β PbO QD/PS-composite-film-based SA at 1064 nm. (**c**) Schematic diagram of the YDFL with the PbO QD/PS-composite-film-based SA. (**d**) Output power versus pump power. (**e**) Long-term stability under water immersion for 70 min. (**f**) The shifts of the central wavelength and 3 dB bandwidth within 70 min, when the pigtails were completely immersed in water. Inset shows the photograph of the fiber pigtails under water immersion. Reproduced with permission from Reference [17]. Copyright 2019, The Royal Society of Chemistry.

In addition, our group successfully fabricated for the first time a novel nanocomposite film composed of PbO QDs and polystyrene (PS) by solution blending for ultrafast photonics in harsh conditions such as marine environments [17]. The as-fabricated PbO QDs with an average diameter and thickness of 4.8 ± 1.1 nm and 2.3 ± 0.5 nm, respectively, are well distributed in the PbO QD/PS nanocomposite film, and the as-obtained composite film is transparent and flexible without apparent bubbles or other physical defects that will severely affect the device performance in electronics and photonics. The open aperture *Z*-scan test of the as-prepared composite film was carried out to investigate the performance after water immersion (Figure 10a,b). Before and after the water immersion, a stable nonlinear absorption at 1064 nm was obtained (Figure 10a). The nonlinear transmission, which strongly depends on the incident laser intensity, was obtained by extracting the *Z*-scan results (Figure 10b). This combines with the nonlinear properties observed in the pure PS film, indicating that the nonlinear absorption effect indeed originates from the PbO QD/PS nanocomposite film. To evaluate the mode-locking performance, the as-prepared PbO QD/PS nanocomposite film employed as a SA was incorporated into the ring cavity of an ytterbium-doped fiber laser (YDFL) (Figure 10c). It can be observed that the laser output power has a linear relationship with the pump power in the range from 55 mW to 180 mW (Figure 10d). Note that the threshold power for the mode-locking pulse generation based on the PbO QD/PS nanocomposite film is significantly lower than those of other SA-based YDFLs, such as MoS_2_ [97], MXene Ti_3_C_2_T*_x_* [98], perovskite CH_3_NH_3_PbI_3_ NSs [99], and the few-layer Bi [100], suggesting that a mode-locking state with a relatively lower input power can be easily achieved. The long-term measurement of fiber lasers based on the PbO QD/PS nanocomposite film in water exhibits that the output spectrum slightly changes in the beginning and then remains relatively stable for at least 70 min (Figure 10e). It should be pointed out here that the life-time of the artificial PbO/PS-nanocomposite-film-based SAs mainly depends on the aging of the optical irradiation due to the PbO photocorrosion [20,92] and working temperature because of the relatively low glass temperature [101]. The evolution of the central wavelength illustrates that the central wavelength drifts from 1062.84 nm to 1063.78 nm, and the 3-dB spectral bandwidth presents a weak perturbation from 1.52 nm to 1.90 nm (Figure 10f), both of which confirm the excellent water resistance of the as-prepared PbO QD/PS nanocomposite film. It can also be observed that the as-prepared PbO QD/PS nanocomposite film has a good stability in water (Figure 10f inset), which is attributed to the excellent insolubility of the PS and β-PbO QDs. Based on this study, it is expected that the PbO-based nanocomposite can be employed as an alternative SA for new designs of a stable mode-locked YDFL with versatile functionalities, such as flexibility, waterproofness, self-cleaning, self-healing, etc.

## 4. Conclusions and Outlook

In the past twenty years, black-phosphorus-analog PbO has achieved great progress due to its versatile advantages, such as its low cost, facile fabrication, high surface area, suitable bandgap, and good radiation-absorbing properties. In this review, PbO nanostructures with different dimensionalities were summarized in detail, and their fundamental properties and optoelectronics/photonics applications were also reviewed. However, although the application prospects of PbO nanostructures in nanophotonics are interesting and promising, the exploration of PbO nanostructures in the field of optoelectronics/photonics is still relatively new compared to that of other black phosphorus analogs, such as Te [13,14], Se [6], Bi [4], antimony [12], etc. Firstly, it is crucial to take the mass production and process integration of PbO nanostructures into consideration for the development of low-cost, facile, and ecofriendly synthetic methodologies to meet the demands of industrial application. Secondly, functional PbO nanostructures, such as doped PbO or PbO-based heterostructures, which are capable of significantly improving the performance of PbO nanomaterials, are a research hotspot. Thirdly, the multifunctional performance based on PbO nanostructures (surface-modified 2D PbO, 2D PbO/polymer, etc.) for multidisciplinary applications, such as photodetection with a self-cleaning ability and nonlinear photonics with a self-healing ability, is a burgeoning field because of the superior properties in each disparate component.

## Data Availability

Not applicable.
